# Supplementary material for: Essential role of FBXL5-mediated cellular iron homeostasis in maintenance of hematopoietic stem cells
Source: Nat Commun. 2017 Jul 17;8:16114. doi: 10.1038/ncomms16114 (PMC5520054; doi:10.1038/ncomms16114)
Supplement: Supplementary Information [file ncomms16114-s1.pdf]

File name: Supplementary Information

Description: Supplementary Figure

File name: Supplementary Data 1

Description:

Differentially expressed genes (P value of <0.05) up-regulated in Fbxl5-KO HSCs compared with control cells.

File name: Supplementary Data 2

Description: **Differentially expressed genes (P value of <0.05) down-regulated in Fbxl5-KO HSCs compared with control cells.**

File name: Peer Review File

Description:

Supplementary Figure Legends

Supplementary Figure 1 | FBXL5 mRNA is expressed in hematopoietic cells including HSCs. (**a**) RT and real-time PCR analysis of FBXL5 mRNA in various hematopoietic cell types of wild-type mice (n = 3). Data are means + s.d. MPP, multipotent progenitors (CD150^{-}KSL cells); CMP, common myeloid progenitors (c-Kit^{+}Sca-1^{-}Lin^{-} CD34^{+} CD16/32^{low} cells); GMP, granulocyte-macrophage progenitors (c-Kit^{+}Sca-1^{-}Lin^{-} CD34^{+} CD16/32^{high} cells); MEP , megakaryocyte-erythrocyte progenitors (c-Kit^{+}Sca-1^{-}Lin^{-} CD34^{-} CD16/32^{low} cells). (**b**, **c**) *FBXL5* expression levels in the mouse hematopoietic system shown as output from Gene Expression Commons (**b**) or from BloodSpot (GSE60101) (**c**).

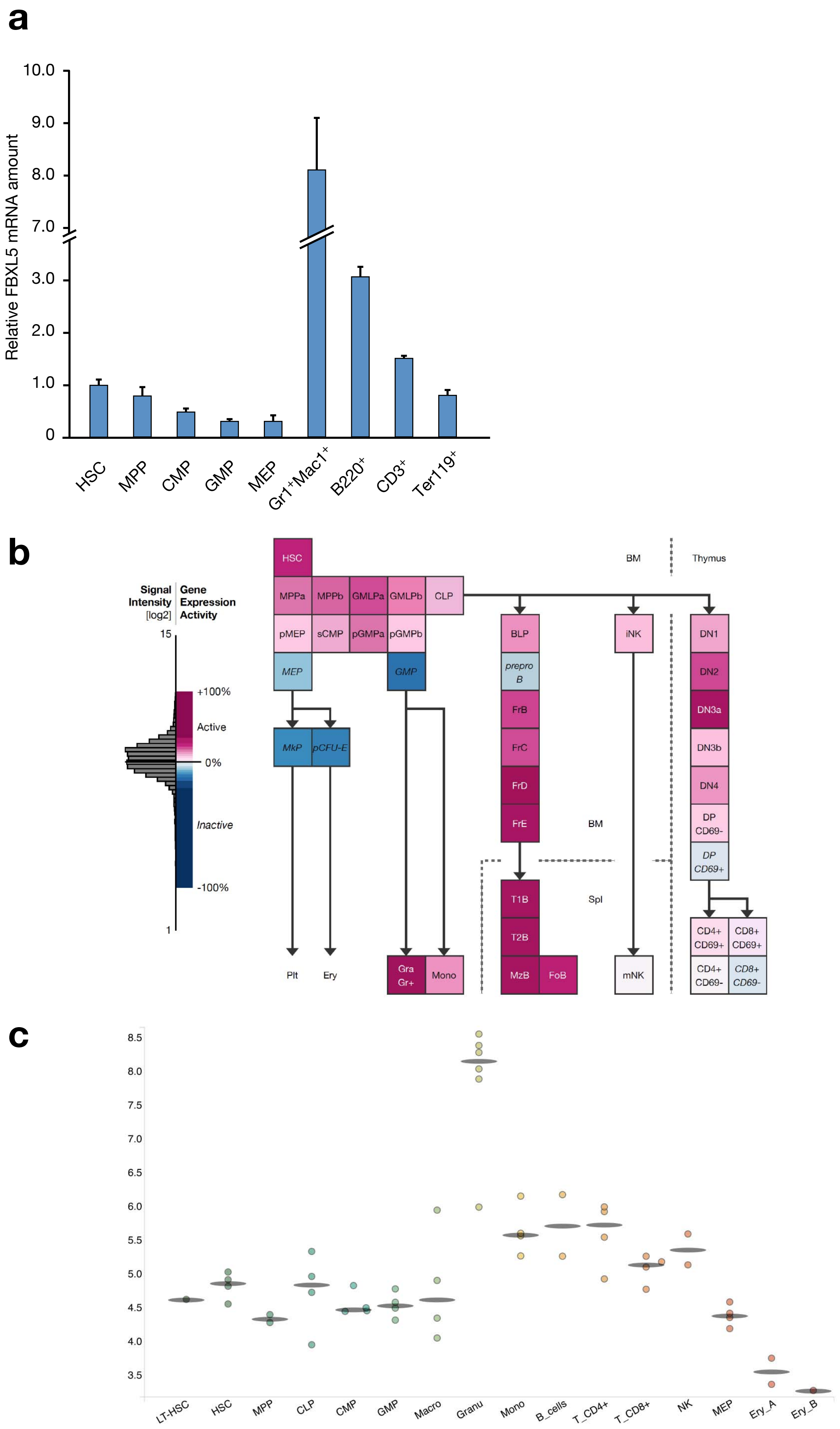

Muto *et al.* Supplementary Figure 1
